# Expert meeting report: epidemiology and management of acquired hypothalamic obesity

**DOI:** 10.3389/fendo.2026.1798619

**Published:** 2026-04-15

**Authors:** Hermann L. Müller, Ute K. Bartels, Christian Denzer, Ulrich Dischinger, Jörg Flitsch, Johannes Gojo, Annette Richter-Unruh, Katrin Scheinemann, Katharina Schilbach, Carsten Friedrich

**Affiliations:** 1 Department of Pediatrics and Pediatric Hematology/Oncology, University Children’s Hospital, Carl von Ossietzky Universität, Klinikum Oldenburg Anstalt des öffentlichen Rechts (AöR), Oldenburg, Germany; 2 Section of Pediatric Brain Tumors, Hopp Children’s Cancer Center Heidelberg, German Cancer Research Center (DKFZ), Heidelberg, Germany; 3 Division of Pediatric Endocrinology and Diabetes, Department of Pediatrics and Adolescent Medicine, University Medical Center Ulm, Ulm, Germany; 4 Department of Internal Medicine, Division of Endocrinology and Diabetes, University Hospital Würzburg, Würzburg, Germany; 5 Department of Neurosurgery, University Medical Center Hamburg-Eppendorf, Hamburg, Germany; 6 Department of Pediatrics and Adolescent Medicine, Comprehensive Center for Pediatrics and Comprehensive Cancer Center, Medical University of Vienna, Vienna, Austria; 7 Hormonzentrum für Kinder und Jugendliche, Überörtliche Berufsausübungsgemeinschaft (ÜBAG), Medizinisches Versorgungszentrum (MVZ) Eberhard & Partner Dortmund, Dortmund, Germany; 8 Division of Pediatric Hematology and Oncology, Children’s Hospital of Eastern Switzerland, St. Gallen, Switzerland; 9 Faculty of Health Sciences and Medicine, University of Lucerne, Lucerne, Switzerland; 10 Medizinische Klinik und Poliklinik IV, Ludwig-Maximilians-University (LMU) Klinikum München, Munich, Germany

**Keywords:** craniopharyngioma, hypothalamus, irradiation, neurosurgery, obesity, quality of life, semaglutide, setmelanotide

## Abstract

Acquired hypothalamic obesity (aHO) is a disease characterized by rapid, clinically significant, and persistent weight gain resulting from damage to hypothalamic structures. aHO is associated with substantial morbidity, increased mortality, and marked impairment in quality of life. Etiologies include craniopharyngioma and other space-occupying lesions of the sellar/parasellar region, neurosurgical procedures, cranial irradiation, and traumatic brain injury. A multidisciplinary panel comprising ten specialists in neuroendocrinology, neurooncology, and neurosurgery from Germany, Austria, and Switzerland convened in Frankfurt am Main, Germany, on November 10, 2025, to discuss contemporary challenges and advances in this field. aHO should be conceptualized and treated within the broader clinical entity of hypothalamic syndrome, a complex disorder involving multiple neuroendocrine deficiencies, disturbances of circadian regulation, impaired control of hunger, satiety, and thirst, altered thermoregulation, and a range of cognitive, sleep-related, and psychosocial dysfunctions. Long-term outcomes for affected individuals are frequently unfavorable, largely due to increased risks of metabolic syndrome, cardiovascular disease, profound reductions in health-related quality of life, and elevated rates of premature mortality. The management of hypothalamic syndrome remains particularly challenging. Pharmacological strategies, including dextroamphetamine and glucagon-like peptide-1 receptor agonists, have demonstrated potential benefits for weight and hyperphagia-related outcomes. Recently, preliminary findings from a prospective, randomized, placebo-controlled clinical trial (TRANSCEND) provided encouraging evidence for the efficacy of setmelanotide, a melanocortin-4 receptor agonist. This perspectives report reviews clinical advances in epidemiology, diagnostics, treatment, and follow-up of patients with aHO and outlines key directions for future research aimed at improving outcomes in this vulnerable population.

## Introduction

A scientific workshop addressing the disease of acquired hypothalamic obesity (aHO) was held in Frankfurt am Main, Germany, on November 10, 2025. The meeting convened 10 experts in endocrinology, pediatric endocrinology, pediatric oncology, pediatric neurooncology, and neurosurgery. The objectives were: 1) to discuss novel insights on epidemiology of aHO, 2) to derive preliminary, practice-oriented recommendations for diagnosis and management across endocrine and non-endocrine sequelae; 3) to identify priorities, unmet needs and requirements for future research. There was no formal consensus process (no delphi or voting, no grading of recommendations).

aHO is a consequence of hypothalamic damage (1), and is characterized by rapid and severe weight gain that is typically resistant to lifestyle-based interventions (2–4). Conventional lifestyle interventions applied to date have generally resulted in only transient reductions in BMI, suggesting that durable weight control is unlikely to be achieved without continuous support and structured behavioral coaching (5). Achieving long-term treatment success requires a stable and supportive home environment, particularly in light of the complex interaction between persistent hyperphagia, pituitary hormone deficiencies, and behavioral disturbances commonly observed in patients with aHO (6).

**Figure 1:**
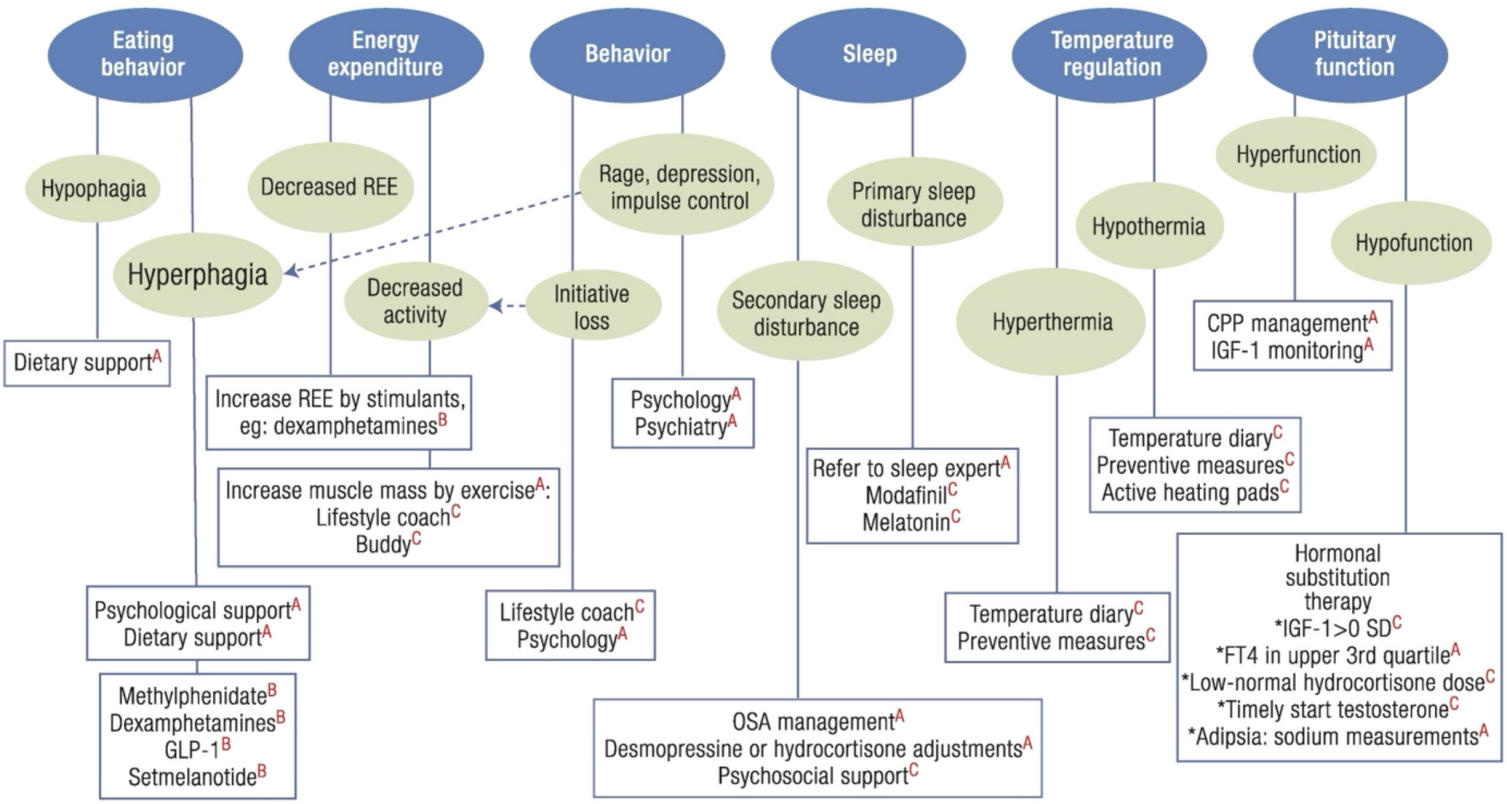
Algorithm for the management of (acquired) hypothalamic dysfunction. Hypothalamic dysfunction may lead to different signs and symptoms that may all contribute to the development of hypothalamic overweight or obesity and feelings of fatigue. By assessing six clinical domains of hypothalamic dysfunction in a stepwise manner, clinicians can individualize management to reduce fatigue and mitigate weight gain. Interventions are categorized as guideline-based **(A)**, supported by trial/case-series data **(B)**, or expert opinion **(C)**. For “Pituitary function” also water balance measurement should be considered as an important parameter in management of arginine vasopressin deficiency. Reproduced from van Santen and Müller, Endocr Rev, 2025 (15), with kind permission of Illustration Presentation ENDOCRINE SOCIETY. CPP, central precocious puberty; OSA, obstructive sleep apnea; REE, resting energy expenditure; IGF, insulin-like growth factor; fT4, free thyroxine; GLP, glucagon-like peptide; SD, standard deviation.

aHO most frequently develops following diagnosis and surgical treatment of craniopharyngioma (7, 8) or other supra- and parasellar tumors. However, aHO may also arise after traumatic brain injury (TBI) or hypothalamic microinjury (9). aHO represents a core manifestation of hypothalamic syndrome, which is defined by dysregulation of the autonomic nervous system (10), hyperinsulinemia, reduced sympathetic tone (11), leptin resistance, altered energy expenditure, and pituitary dysfunction. These abnormalities are commonly accompanied by sleep–wake disturbances (12, 13), neurological and visual impairments (14), and reduced physical activity (15, 16) (**Figure 1**). Hypothalamic involvement in craniopharyngioma is a key determinant of long-term outcomes, particularly regarding weight development, quality of life (17) and overall survival (18, 19).

Pharmacological treatment approaches, including central nervous system stimulants (e.g., dextroamphetamine) (20, 21) have shown potential benefits for weight and hyperphagia-related outcomes. Glucagon-like peptide-1 receptor (GLP-1R) agonists – as monotherapy or in combination with other agents - seem also promising in the treatment of aHO by bypassing damaged hypothalamic pathways and reducing appetite via preserved brainstem and peripheral satiety signaling (22). Emerging evidence suggests that GLP-1R agonists modulate the mesocorticolimbic reward circuitry by acting on receptors within the ventral tegmental area (VTA) and the nucleus accumbens (NAc), where they attenuate dopaminergic signaling and reduce the reinforcing value of palatable food (23).

A small study of 26 adults with aHO reported weight loss in all but one participant after semaglutide treatment, with a mean reduction of -13.4 kg and a BMI decrease of -4.4 kg/m² at one year (24). Recent case reports in patients with aHO following craniopharyngioma indicate that semaglutide is a safe therapy associated with sustained long-term improvements in eating behavior, weight regulation, metabolic parameters (25), and quality of life, persisting after weight stabilization (26). Consistent with these observations, a systematic review of 10 studies concluded that GLP-1 receptor agonists may represent a safe and effective strategy for weight management in aHO (27). Nevertheless, their efficacy in adults remains controversial. Some studies reported enhanced glycemic control and reduced food intake (27, 28), whereas Shoemaker et al. (28) described a paradoxical decline in total energy expenditure following weekly exenatide administration, disproportionate to weight loss and unrelated to physical activity or leptin changes. In contrast, Perez et al. (29) found greater adiposity reduction at the same exenatide dose in patients with more extensive hypothalamic injury. Overall, preliminary data suggest that semaglutide and exenatide may offer therapeutic benefit for weight management in aHO (22).

Recently, preliminary findings from a prospective, randomized, placebo-controlled clinical trial (TRANSCEND, NCT05774756) enrolling 120 individuals with aHO provided encouraging evidence for the efficacy of setmelanotide, a melanocortin-4 receptor (MC4R) agonist (30). After 52 weeks of treatment, participants receiving setmelanotide (n = 81) achieved a mean reduction in body mass index (BMI) of 16.5% (p <0.0001), whereas those assigned to placebo (n = 39) experienced a mean BMI increase of 3.3%. Notably, 80% of individuals in the active treatment group attained a BMI reduction of at least 5% by week 52. These results indicate that setmelanotide may constitute a promising therapeutic option for the treatment of aHO (31). Setmlanotide acts on the MC4 receptors in the hypothalamus to re-establish the impaired pathway and activate satiety and leads to weight loss. Given that hypothalamic injury may compromise hypothalamic integrity in aHO, the mechanism by which such treatment could exert benefit warrants careful consideration. Importantly, in many cases of aHO, hypothalamic damage is partial rather than complete, resulting in residual hypothalamic function. Under these circumstances, setmelanotide may activate remaining hypothalamic neurons and partially restore satiety signaling. Siljee et al. could show that the expression of MC4R mRNA in the human hypothalamus is widespread and in close approximation to endogenous MC4R binding partners Agouti-related peptide (AgRP) and alpha-melanocyte-stimulating hormone (α-MSH). Most intense MC4R mRNA expression was present in the paraventricular nucleus (PVN), the supraoptic nucleus (SON), and the nucleus basalis of Meynert. Most MC4R-positive cells in the SON also expressed arginine vasopressin/oxytocin (32). Moreover, MC4R expression has been demonstrated not only within the hypothalamus but also in extra-hypothalamic regions, including the cerebral cortex and spinal cord, suggesting potential sites of action beyond the hypothalamus itself.

## Epidemiology and socioeconomic burden

**Figure 2:**
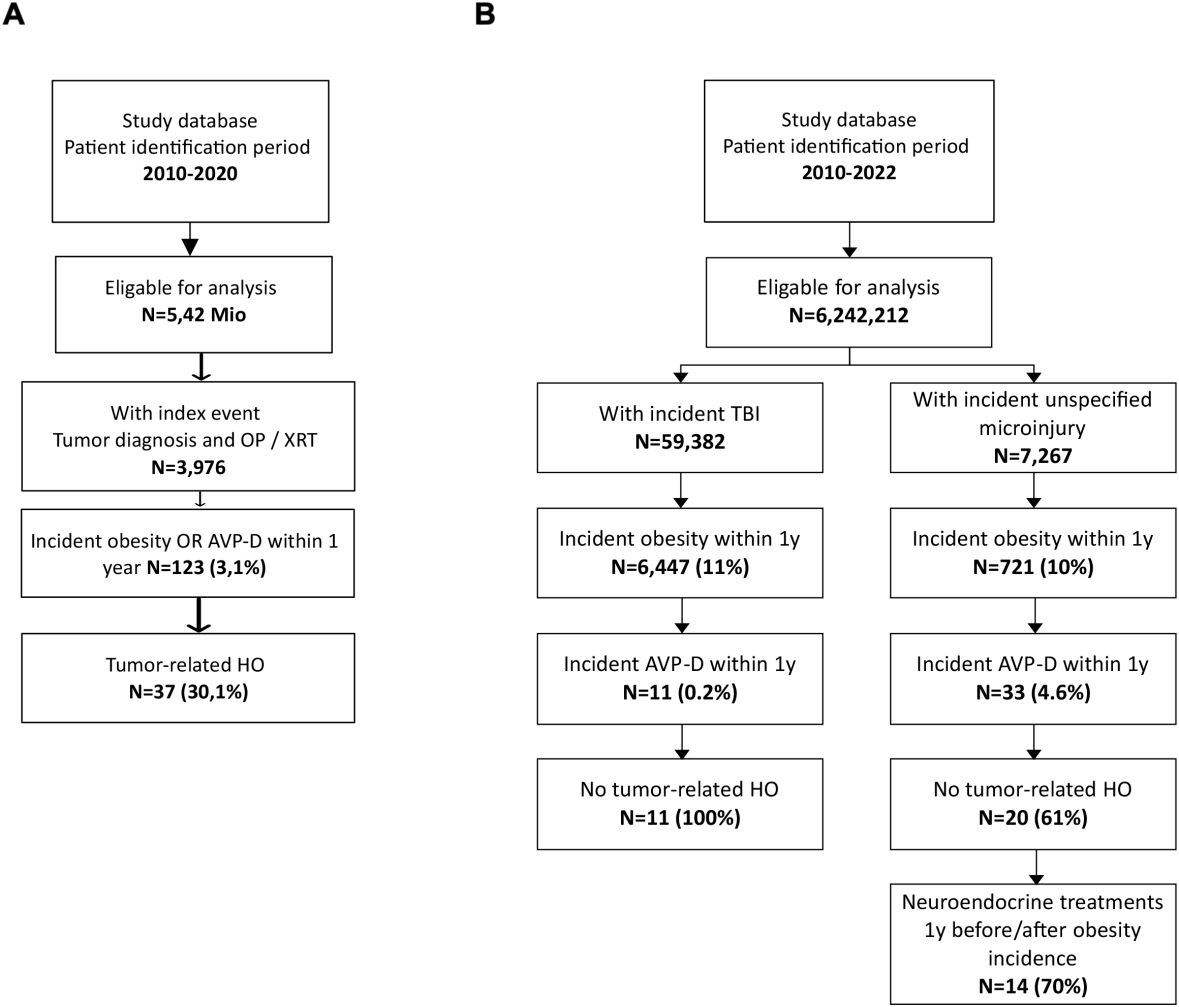
Epidemiological results of German claims data analyses (collected between 2010 and 2020) for **(A)** tumor- or treatment-related, acquired hypothalamic obesity (aHO) (33) and **(B)** aHO in patients with traumatic brain injury (TBI) and unspecified hypothalamic microinjury (UM) (data collected between 2010 and 2022). Modified and reproduced from Witte et al., 2025, J Neuroendocrinol (36), with kind permission of Wiley. aHO, acquired hypothalamic obesity; AVP-D, arginine vasopressin deficiency; y, years; XRT, irradiation; OP, surgery; TBI, traumatic brain injury; Mio, million.

The epidemiology and socioeconomic burden of aHO were evaluated using German statutory health insurance claims data (33, 34). The analysis was based on 5.42 million insured individuals with at least 24 months of continuous data between 2010 and 2020. After applying wash-in periods to exclude individuals with prior tumor-related brain surgery, radiotherapy, obesity, or arginine vasopressin deficiency (AVP-D), a cleaned cohort of 3,976 individuals remained. The index event was defined as hospitalization for a tumor associated with aHO combined with tumor-related neurosurgery or radiotherapy. aHO was identified when incident obesity occurred at the index hospitalization or within 12 months thereafter and was validated by the presence of AVP-D and/or desmopressin treatment. Using these criteria, 37 patients fulfilled the claims-based definition of aHO (**Figure 2A**). This corresponds to an estimated annual incidence of 0.07 – 0.17 per 100,000 individuals, or approximately 80 new cases per year in Germany, with a prevalence of 1,262 cases in 2019. Age distribution included 203 patients <20 years, 784 patients aged 20–64 years, and 275 patients >65 years of age. Benign sellar tumors, predominantly craniopharyngioma, accounted for approximately half of all cases. Claims-based case definitions may misclassify obesity onset and cannot capture key clinical features such as hyperphagia, resting energy expenditure, or MRI-based hypothalamic involvement, likely leading to a certain under-ascertainment.

Healthcare resource utilization analysis demonstrated that patients with aHO required substantially more healthcare services than BMI-matched controls (35). In the first year following the index event, patients with aHO experienced an average of 4.6 additional hospitalizations, with excess hospital use persisting into the second year. Index-quarter costs were markedly higher in patients with aHO (€25,717) compared with controls (€886), largely driven by tumor-related hospitalizations. Excess annual costs relative to controls were €19,935 in the first year and €10,745 in the second year after the index event, with long-term endocrine replacement therapy constituting a major cost driver.

In a comprehensive aHO cohort (data collected between 2010 and 2022) (36), the role of TBI and hypothalamic microinjury was further analyzed (**Figure 2B**). Among more than 6.2 million insured individuals, 59,382 incident TBIs were identified, of whom 11% developed obesity within one year. None of the TBI patients presented with intracranial space-occupying lesions. Only a small subset (11 patients) developed confirmed aHO with documented AVP-D or pituitary dysfunction, none of which were tumor-related. Among 7,267 individuals with unspecified hypothalamic microinjury, 10% developed obesity within one year, and 4.6% exhibited incident AVP-D. After excluding tumor-related cases, 20 patients remained, the majority of whom received neuroendocrine treatment around the time of obesity onset.

Overall, an estimated 2,500 cases of aHO were present in 2019/2020. Sixty percent were tumor-related, 22% were associated with unspecified microinjury, and 18% with TBI. Tumor-related aHO predominantly affected females (59%), whereas TBI-associated aHO was more common in males (73%). Participants of the workshop noted that these estimates – calculated with methods, designed to avoid false positive results – likely underestimate the true prevalence of aHO, particularly in adults. In addition, rare conditions such as ROHHAD syndrome (Rapid-onset obesity with hypothalamic dysfunction) may also remain underdiagnosed as cause of aHO (37, 38).

## Diagnostics and management

Reduced growth velocity and/or headache and visual disturbances should be regarded as an early warning sign of craniopharyngioma in childhood (39, 40). Despite its clinical severity, aHO remains under-recognized and difficult to diagnose due to the absence of standardized biomarkers, diagnostic tests, and comprehensive clinical guidelines (41, 42). Recently proposed international diagnostic guidelines for aHO require fulfillment of three criteria (43):

1. A documented traumatic event or neurooncologic disease (causing hypothalamic damage visible on magnetic resonance imaging (MRI));
2. Clinically relevant (≥5% BMI increase in adult; ≥+1.0 SDS BMI increase in pediatric patients), rapid and persistent (for 24 months after surgery) BMI increase beginning within 12 months after the index event, under clinical and anthropometric monitoring at 3 months intervals; and
3. Attainment of age- and ethnicity-adjusted overweight or obesity thresholds (BMI SDS ≥+2.0 SD in pediatric patients; BMI ≥25 kg/m^2^ or BMI ≥30 kg/m^2^ in adult patients, depending on racial and ethnic characteristics).

In parallel, a seven-domain scoring system for hypothalamic syndrome has been introduced (10, 44), encompassing BMI changes, hyper-/hypophagia, behavioral disturbances, sleep abnormalities, temperature dysregulation, pituitary deficits, and neuroimaging findings. However, incomplete documentation of sleep, behavioral, and thermoregulatory features in routine care highlights significant gaps in current clinical practice. Earlier diagnosis may be facilitated by standardized post-surgical follow-up protocols that include regular anthropometric assessments, temperature monitoring, and targeted questionnaires addressing eating behavior, fatigue, and sleep disturbances. Rehabilitation programs, particularly in pediatric populations, may provide a structured setting for standardized assessment and intervention. However, long-term effects of rehabilitation programs on weight development are limited (45). A planned research initiative at the University of Oldenburg and the University Hospital Würzburg will evaluate real-world outcomes of different therapeutic strategies for aHO. Ultimately, evidence-based diagnostic tools and criteria are needed to support future guideline development.

## aHO - a distinct disease entity

Similar to central hypothyroidism, growth hormone deficiency, central hypogonadism, and central hypocortisolism, and comparable to non-endocrine chronic diseases such as chronic renal failure, aHO should be recognized as a distinct clinical disorder resulting from hypothalamic dysfunction, with consistent clinical features and specific therapeutic needs. Formal recognition as a distinct disease would facilitate standardized coding, access to multidisciplinary care pathways, and eligibility for clinical trials and emerging targeted therapies. Regardless of etiology, participants agreed that treatment goals include improvement of eating behavior, increasing energy expenditure to achieve weight stabilization or loss, improving body composition, reducing cardiovascular risk, optimizing neuroendocrine replacement therapy, improving circadian rhythm disturbances, and enhancing psychosocial functioning. Collectively, these measures are expected to result in meaningful improvements in quality of life.

## Surgical strategy and expertise

Surgical approach, institutional case volume, and neuro-oncologic expertise have been identified as key prognostic factors for the development of aHO following craniopharyngioma treatment (46). Iatrogenic hypothalamic injury is associated with increased risk of aHO and reduced quality of life (17). Surgical strategy is strongly influenced by tumor location, patient age, and surgeon experience. Larger centers more frequently employ hypothalamus-sparing techniques, and patient load reflecting center size and expertise has emerged as an independent prognostic factor for aHO risk (47–49). Participants anticipated that mandatory certification of neurosurgical centers and further centralization of craniopharyngioma care over the next decade is expected to be associated with improved patient outcomes. Experience from the Netherlands suggests that centralized treatment in specialized centers is associated with improved outcomes for patients with craniopharyngioma (50).

## Bariatric interventions

Bariatric surgical interventions have demonstrated substantial efficacy and acceptable safety profiles in facilitating weight reduction among patients with craniopharyngioma. In an individual patient–level meta-analysis comprising 21 CP cases, Bretault et al. (51) reported significant postoperative weight loss at both 6 and 12 months, with percentage total weight loss of −0.9% and −15.1%, respectively. The greatest degree of weight reduction was observed in patients undergoing Roux-en-Y gastric bypass procedures. In pediatric cohorts, the application of irreversible bariatric techniques, including gastric bypass, remains ethically and legally controversial and should preferably be restricted to controlled clinical trial settings (52). According to the Endocrine Society’s Clinical Practice Guideline for the management of pediatric obesity, bariatric surgery should be considered only in adolescents with severe obesity who have reached advanced pubertal development and near-final or final adult stature, and who have demonstrated sustained adherence to structured dietary and physical activity interventions (53).

## Patient cases

Setmelanotide, a MC4R agonist, represents a mechanism-based therapeutic approach targeting satiety pathways downstream of leptin signaling (54). Phase 3 data from the TRANSCEND study (7) along with clinical and real-world case reports from several German centers, illustrated the potential efficacy of setmelanotide in aHO. Treatment effects were primarily described as restoration of physiological satiety. Skin hyperpigmentation, the most common adverse effect, occurred in most patients but was generally well tolerated. Participants emphasized the need for structured safety monitoring and standardized patient-reported outcomes on hunger/satiety and quality of life in real-world use. Discussion suggested that additional patient subgroups with undetected MC4R-related dysfunction may exist, including individuals with TBI, hypothalamic microinjury, or rare inflammatory events such as ROHHAD syndrome (10), who develop rapid weight gain and panhypopituitarism in the absence of tumor surgery (36).

## Results and key clinical conclusions

Craniopharyngioma: Hypothalamus-sparing surgical strategies should be prioritized to prevent aHO. Long-term outcomes are largely determined by late sequelae, particularly aHO; therefore, management should be conducted within experienced multidisciplinary teams. There is a critical need for policy-level initiatives aimed at defining and implementing quality criteria for multidisciplinary craniopharyngioma management (e.g., minimum case volume, multidisciplinary tumor boards with neuroendocrinology, standardized postoperative follow-up including hypothalamic syndrome screening). Future investigations should extend beyond radiological endpoints to rigorously assess the impact of evolving surgical and medical therapies on cognitive function, cardiometabolic morbidity, hypothalamic morbidity, and overall patient well-being in the context of national or international registries and trials.

Other brain tumors: A range of histological tumor entities, including low-grade glioma and germ-cell tumors, can occur in the sellar region and therefore require different surgical and oncological strategies. Despite this heterogeneity, hypothalamic damage has been reported and may still be underdiagnosed in this patient group. In analogy to ongoing efforts in craniopharyngioma, a structured evaluation of surgical, radiotherapeutic, and oncological approaches—together with a systematic assessment of aHO and the broader hypothalamic syndrome—is essential to better quantify disease burden and to inform future therapeutic strategies in this population.

aHO/Hypothalamic syndrome: Recognizing aHO as a distinct clinical entity, defined by functional hypothalamic disruption rather than solely by etiology, is essential for advancing research, improving diagnosis and care, and expanding access to emerging therapies. There is a critical need for further research into targeted therapies addressing aHO and its underlying neuroendocrine and behavioral mechanisms (8). Future studies should systematically include patients with non-tumor etiologies, such as TBI, SOD, ROHHAD, and other midline or intracranial pathologies. Standardized diagnostic criteria and outcome measures for aHO (including standardized hunger/satiety metrics, sleep/circadian measures, resting energy expenditure (REE)/physical activity assessments, and health-related quality of life instruments) are essential to enable meaningful comparisons across studies and clinical trials. Determination of the most effective timing (early vs. delayed initiation) and integration of novel targeted therapies with surgery and radiotherapy is warranted.

## Data Availability

The original contributions presented in the study are included in the article/Supplementary Material. Further inquiries can be directed to the corresponding author.

## References

[1] Gan HW Cerbone M Dattani MT . Appetite- and weight-regulating neuroendocrine circuitry in hypothalamic obesity. Endocr Rev. (2024) 45:309–42. doi: 10.1210/endrev/bnad033. PMID: 38019584 PMC11074800

[2] Muller HL . Management of acquired hypothalamic obesity after childhood-onset craniopharyngioma-a narrative review. Biomedicines. (2025) 13. doi: 10.3390/biomedicines13051016, PMID: 40426846 PMC12109346

[3] van Iersel L Brokke KE Adan RAH Bulthuis LCM van den Akker ELT van Santen HM . Pathophysiology and individualized treatment of hypothalamic obesity following craniopharyngioma and other suprasellar tumors: a systematic review. Endocr Rev. (2019) 40:193–235. doi: 10.1530/ey.16.11.14. PMID: 30247642

[4] Van Roessel I Van Den Brink M Dekker J Ruitenburg-van Essen BG Tissing WJE van Santen HM . Feasibility, safety, and efficacy of dietary or lifestyle interventions for hypothalamic obesity: a systematic review. Clin Nutr. (2024) 43:1798–811. doi: 10.1016/j.clnu.2024.05.028. PMID: 38955055

[5] Rakhshani N Jeffery AS Schulte F Barrera M Atenafu EG Hamilton JK . Evaluation of a comprehensive care clinic model for children with brain tumor and risk for hypothalamic obesity. Obes (Silver Spring). (2010) 18:1768–74. doi: 10.1038/oby.2009.491. PMID: 20057368

[6] Meijneke RW Schouten-van Meeteren AY de Boer NY van Zundert S van Trotsenburg PA Stoelinga F . Hypothalamic obesity after treatment for craniopharyngioma: the importance of the home environment. J Pediatr Endocrinol Metabol: JPEM. (2015) 28:59–63. doi: 10.1515/jpem-2014-0338. PMID: 25381948

[7] Muller HL . Craniopharyngioma. Endocr Rev. (2014) 35:513–43. doi: 10.1530/endoabs.35.s4.1. PMID: 24467716

[8] Muller HL . Craniopharyngioma - what's next. Pituitary. (2025) 28:125. doi: 10.1093/neuonc/nou067. PMID: 41205018

[9] Muller HL Tauber M Lawson EA Ozyurt J Bison B Martinez-Barbera JP . Hypothalamic syndrome. Nat Rev Dis Primers. (2022) 8:24. doi: 10.1530/endoabs.110.ep1084. PMID: 35449162

[10] Doelman-Oldenburger NJ Muller HL van Santen HM . Novel autonomic dysregulation score in children with hypothalamic syndrome. Endocr Connect. (2025) 14. doi: 10.1530/endoabs.110.ep1084. PMID: 41251290 PMC12673360

[11] Roth CL Hunneman DH Gebhardt U Stoffel-Wagner B Reinehr T Muller HL . Reduced sympathetic metabolites in urine of obese patients with craniopharyngioma. Pediatr Res. (2007) 61:496–501. doi: 10.1055/s-2006-974094. PMID: 17515878

[12] Muller HL . Sleep disorders, dysregulation of circadian rhythms, and fatigue after craniopharyngioma-a narrative review. Biomedicines. (2025) 13. doi: 10.3390/biomedicines13102356, PMID: 41153643 PMC12561223

[13] Mann-Markutzyk LV Beckhaus J Ozyurt J Mehren A Friedrich C Muller HL . Daytime sleepiness and health-related quality of life in patients with childhood-onset craniopharyngioma. Sci Rep. (2025) 15:9407. doi: 10.1530/endoabs.99.p329. PMID: 40108339 PMC11923165

[14] Sowithayasakul P Beckhaus J Boekhoff S Friedrich C Calaminus G Muller HL . Vision-related quality of life in patients with childhood-onset craniopharyngioma. Sci Rep. (2023) 13:19599. doi: 10.1038/s41598-023-46532-y. PMID: 37949931 PMC10638396

[15] van Santen HM Muller HL . Management of acquired hypothalamic dysfunction and the hypothalamic syndrome; it is more than obesity. Endocr Rev. (2025) 46(6):891–907. doi: 10.1210/endrev/bnaf025. PMID: 40746184 PMC12640713

[16] Harz KJ Muller HL Waldeck E Pudel V Roth C . Obesity in patients with craniopharyngioma: assessment of food intake and movement counts indicating physical activity. J Clin Endocrinol Metab. (2003) 88:5227–31. doi: 10.1210/jc.2002-021797. PMID: 14602754

[17] Bogusz A Boekhoff S Warmuth-Metz M Calaminus G Eveslage M Muller HL . Posterior hypothalamus-sparing surgery improves outcome after childhood craniopharyngioma. Endocr Connect. (2019) 8:481–92. doi: 10.1530/ec-19-0074. PMID: 30925462 PMC6479199

[18] Sterkenburg AS Hoffmann A Gebhardt U Warmuth-Metz M Daubenbuchel AM Muller HL . Survival, hypothalamic obesity, and neuropsychological/psychosocial status after childhood-onset craniopharyngioma: newly reported long-term outcomes. Neuro-oncology. (2015) 17:1029–38. doi: 10.1055/s-0035-1547724. PMID: 25838139 PMC5654354

[19] Beckhaus J Friedrich C Boekhoff S Calaminus G Bison B Eveslage M . Outcome after pediatric craniopharyngioma: the role of age at diagnosis and hypothalamic damage. Eur J Endocrinol Eur Fed Endocrine Societies. (2023) 188. doi: 10.1093/ejendo/lvad027. PMID: 36857103

[20] van Schaik J Welling MS de Groot CJ van Eck JP Juriaans A Burghard M . Dextroamphetamine treatment in children with hypothalamic obesity. Front Endocrinol. (2022) 13:845937. doi: 10.3389/fendo.2022.845937. PMID: 35355559 PMC8959487

[21] Denzer C Denzer F Lennerz BS Vollbach H Lustig RH Wabitsch M . Treatment of hypothalamic obesity with dextroamphetamine: a case series. Obes Facts. (2019) 12:91–102. doi: 10.1159/000495851. PMID: 30844799 PMC6465734

[22] Roth CL Perez FA Whitlock KB Elfers C Yanovski JA Shoemaker AH . A phase 3 randomized clinical trial using a once-weekly glucagon-like peptide-1 receptor agonist in adolescents and young adults with hypothalamic obesity. Diabetes Obes Metab. (2021) 23:363–73. doi: 10.1111/dom.14224. PMID: 33026160 PMC7821019

[23] Colvin KJ Killen HS Kanter MJ Halperin MC Engel L Currie PJ . Brain site-specific inhibitory effects of the GLP-1 analogue exendin-4 on alcohol intake and operant responding for palatable food. Int J Mol Sci. (2020) 21. doi: 10.3390/ijms21249710. PMID: 33352692 PMC7766977

[24] Svendstrup M Rasmussen AK Kistorp C Klose M Andreassen M . Semaglutide treatment of hypothalamic obesity - a real-life data study. Pituitary. (2024) 27:685–92. doi: 10.1007/s11102-024-01429-5. PMID: 39120810 PMC11513754

[25] Gjersdal E Larsen LB Ettrup KS Vestergaard P Nielsen EH Karmisholt JS . Semaglutide as a promising treatment for hypothalamic obesity: a six-month case series on four females with craniopharyngioma. Pituitary. (2024) 27:723–30. doi: 10.1007/s11102-024-01426-8. PMID: 39088138 PMC11513775

[26] Gjersdal E Klit FO Schmidt Ettrup K Vestergaard P Nielsen EH Vistisen KN . Semaglutide treatment in hypothalamic obesity: two-year outcomes on body composition, appetite, and quality of life. Pituitary. (2025) 28:93. doi: 10.1007/s11102-025-01564-7. PMID: 40830718 PMC12364737

[27] Ng VWW Gerard G Koh JJK Loke KY Lee YS Ng NBH . The role of glucagon-like peptide 1 receptor agonists for weight control in individuals with acquired hypothalamic obesity-a systematic review. Clin Obes. (2024) 14:e12642. doi: 10.1111/cob.12642. PMID: 38273176

[28] Shoemaker AH Silver HJ Buchowski M Slaughter JC Yanovski JA Elfers C . Energy balance in hypothalamic obesity in response to treatment with a once-weekly GLP-1 receptor agonist. Int J Obes (Lond). (2022) 46:623–9. doi: 10.1530/ey.19.11.14. PMID: 34975146 PMC8881399

[29] Perez FA Elfers C Yanovski JA Shoemaker AH Abuzzahab MJ Roth CL . MRI measures of hypothalamic injury are associated with glucagon-like peptide-1 receptor agonist treatment response in people with hypothalamic obesity. Diabetes Obes Metab. (2021) 23:1532–41. doi: 10.1111/dom.14366. PMID: 33651438 PMC8353597

[30] Available online at: https://ir.rhythmtx.com/news-releases/news-release-details/rhythmpharmaceuticals-announces-pivotal-phase-3-transcend-trial (Accessed January 26, 2026).

[31] van Santen HM Denzer C Muller HL . Could setmelanotide be the game-changer for acquired hypothalamic obesity? Front Endocrinol. (2023) 14:1307889. doi: 10.3389/fendo.2023.1307889. PMID: 38239988 PMC10794340

[32] Siljee JE Unmehopa UA Kalsbeek A Swaab DF Fliers E Alkemade A . Melanocortin 4 receptor distribution in the human hypothalamus. Eur J Endocrinol Eur Fed Endocrine Societies. (2013) 168:361–9. doi: 10.1530/eje-12-0750. PMID: 23211571

[33] Witte J Surmann B Batram M Weinert M Flume M Touchot N . Hypothalamic obesity: epidemiology in rare sellar/suprasellar tumors-a German claims database analysis. J Neuroendocrinol. (2024) 36:e13439. doi: 10.1111/jne.13439. PMID: 39191454 PMC11646665

[34] Muller HL Witte J Surmann B Batram M Braegelmann K Flume M . Treatment of patients with tumor/treatment-related hypothalamic obesity in the first two years following surgical treatment or radiotherapy. Sci Rep. (2025) 15:2118. doi: 10.1530/eje-07-0145. PMID: 39814823 PMC11736136

[35] Witte J Touchot N Surmann B Braegelmann K Flume M Beckhaus J . Economics of hypothalamic obesity in patients with craniopharyngioma and other rare sellar/suprasellar tumors. Eur J Health Econ. (2025) 26:1557–67. doi: 10.1007/s10198-025-01786-3. PMID: 40343652 PMC12618323

[36] Witte J Touchot N Batram M Diekmannshemke J Flume M Muller HL . Epidemiology of acquired hypothalamic obesity following traumatic brain injury and nonspecific hypothalamic microinjury: a nationwide German claims data analysis. J Neuroendocrinol. (2025) 38(1):e70108. doi: 10.1111/jne.70108. PMID: 41236025 PMC12799323

[37] Khaytin I Victor AK Barclay SF Benson LA Slattery SM Rand CM . Rapid-onset obesity with hypothalamic dysfunction, hypoventilation, and autonomic dysregulation (ROHHAD): a collaborative review of the current understanding. Clin Auton Res. (2023) 33:251–68. doi: 10.1007/s10286-023-00936-y. PMID: 37162653

[38] Harvengt J Gernay C Mastouri M Farhat N Lebrethon MC Seghaye MC . ROHHAD(NET) syndrome: systematic review of the clinical timeline and recommendations for diagnosis and prognosis. J Clin Endocrinol Metab. (2020) 105. doi: 10.1530/ey.18.11.9. PMID: 32407531

[39] Muller HL Emser A Faldum A Bruhnken G Etavard-Gorris N Gebhardt U . Longitudinal study on growth and body mass index before and after diagnosis of childhood craniopharyngioma. J Clin Endocrinol Metab. (2004) 89:3298–305. doi: 10.1210/jc.2003-031751, PMID: 15240606

[40] Hoffmann A Boekhoff S Gebhardt U Sterkenburg AS Daubenbuchel AM Eveslage M . History before diagnosis in childhood craniopharyngioma: associations with initial presentation and long-term prognosis. Eur J Endocrinol Eur Fed Endocrine Societies. (2015) 173:853–62. doi: 10.1530/endoabs.41.ep765. PMID: 26392473

[41] Muller HL . Preoperative staging in childhood craniopharyngioma: standardization as a first step towards improved outcome. Endocrine. (2016) 51:1–3. doi: 10.1007/s12020-015-0800-x, PMID: 26582066

[42] Muller HL Reichel J Boekhoff S Warmuth-Metz M Eveslage M Peng J . Low concordance between surgical and radiological assessment of degree of resection and treatment-related hypothalamic damage: results of KRANIOPHARYNGEOM 2007. Pituitary. (2018) 21:371–8. doi: 10.1007/s11102-018-0883-5, PMID: 29589225

[43] Muller HL Tanaka T Hasegawa T Isojima T Mori J Kurosaki M . Diagnostic criteria for acquired hypothalamic obesity - international expert guidance document. Endocrine J. (2025) 73(2):341–53. doi: 10.1507/endocrj.EJ25-0408, PMID: 41224353 PMC12895118

[44] van Santen HM van Schaik J van Roessel I Beckhaus J Boekhoff S Muller HL . Diagnostic criteria for the hypothalamic syndrome in childhood. Eur J Endocrinol Eur Fed Endocrine Societies. (2023) 188. doi: 10.1530/endoabs.90.p686. PMID: 36737045

[45] Sterkenburg AS Hoffmann A Gebhardt U Waldeck E Springer S Muller HL . Childhood craniopharyngioma with hypothalamic obesity - no long-term weight reduction due to rehabilitation programs. Klin Padiatrie. (2014) 226:344–50. doi: 10.1055/s-0034-1387747, PMID: 25431867

[46] Elowe-Gruau E Beltrand J Brauner R Pinto G Samara-Boustani D Thalassinos C . Childhood craniopharyngioma: hypothalamus-sparing surgery decreases the risk of obesity. J Clin Endocrinol Metab. (2013) 98:2376–82. doi: 10.1210/jc.2012-3928. PMID: 23633208

[47] Muller HL Gebhardt U Teske C Faldum A Zwiener I Warmuth-Metz M . Post-operative hypothalamic lesions and obesity in childhood craniopharyngioma: results of the multinational prospective trial KRANIOPHARYNGEOM 2000 after 3-year follow-up. Eur J Endocrinol Eur Fed Endocrine Societies. (2011) 165:17–24. doi: 10.1210/endo-meetings.2010.part3.or2.or36-1. PMID: 21490122

[48] Muller HL Gebhardt U Faldum A Warmuth-Metz M Pietsch T Pohl F . Xanthogranuloma, Rathke's cyst, and childhood craniopharyngioma: results of prospective multinational studies of children and adolescents with rare sellar malformations. J Clin Endocrinol Metab. (2012) 97:3935–43. doi: 10.1530/endoabs.32.p885. PMID: 22969141

[49] Mende KC Kellner T Petersenn S Honegger J Evangelista-Zamora R Droste M . Clinical situation, therapy, and follow-up of adult craniopharyngioma. J Clin Endocrinol Metab. (2020) 105. doi: 10.1210/clinem/dgz043. PMID: 31589293

[50] Van Schaik J Schouten-van Meeteren AYN Vos-Kerkhof E Janssens GO Porro GL Fiocco M . Treatment and outcome of the Dutch childhood craniopharyngioma cohort study: first results after centralization of care. Neuro-oncology. (2023) 25:2250–61. doi: 10.1093/neuonc/noad112. PMID: 37381692 PMC10708930

[51] Bretault M Boillot A Muzard L Poitou C Oppert JM Barsamian C . Clinical review: Bariatric surgery following treatment for craniopharyngioma: a systematic review and individual-level data meta-analysis. J Clin Endocrinol Metab. (2013) 98:2239–46. doi: 10.1210/jc.2012-4184. PMID: 23533238

[52] Muller HL . Bariatric interventions in craniopharyngioma patients-best choice or last option for treatment of hypothalamic obesity? J Clin Endocrinol Metab. (2022) 107:e426–8. doi: 10.1210/clinem/dgab567, PMID: 34331765

[53] Styne DM Arslanian SA Connor EL Farooqi IS Murad MH Silverstein JH . Pediatric obesity-assessment, treatment, and prevention: an endocrine society clinical practice guideline. J Clin Endocrinol Metab. (2017) 102:709–57. doi: 10.1210/jc.2016-2573. PMID: 28359099 PMC6283429

[54] Roth CL Scimia C Shoemaker AH Gottschalk M Miller J Yuan G . Setmelanotide for the treatment of acquired hypothalamic obesity: a phase 2, open-label, multicentre trial. Lancet Diabetes Endocrinol. (2024) 12:380–9. doi: 10.1016/s2213-8587(24)00087-1. PMID: 38697184

